# Recovery of Background Structures in Nanoscale Helium Ion Microscope Imaging

**DOI:** 10.6028/jres.119.030

**Published:** 2014-12-31

**Authors:** Alfred S Carasso, András E Vladár

**Affiliations:** National Institute of Standards and Technology, Gaithersburg, MD 20899

**Keywords:** adaptive histogram equalization, background recovery, composed fast-scan frames, HIM, nanoscale helium ion microscopy, noise reduction, progressive fractional diffusion smoothing, SEM

## Abstract

This paper discusses a two step enhancement technique applicable to noisy Helium Ion Microscope images in which background structures are not easily discernible due to a weak signal. The method is based on a preliminary adaptive histogram equalization, followed by ‘slow motion’ low-exponent Lévy fractional diffusion smoothing. This combined approach is unexpectedly effective, resulting in a companion enhanced image in which background structures are rendered much more visible, and noise is significantly reduced, all with minimal loss of image sharpness. The method also provides useful enhancements of scanning charged-particle microscopy images obtained by composing multiple drift-corrected ‘fast scan’ frames. The paper includes software routines, written in *Interactive Data Language* (IDL),^1^ that can perform the above image processing tasks.

## 1. Introduction

The advantages of Helium ion microscopy (HIM) over conventional scanning electron microscopy (SEM) are explored in [9] and [10]. HIM imaging achieves higher resolution with higher contrast and greater depth of field than is possible with SEM. In many cases, HIM images reveal greater surface detail. The secondary electrons that carry the sample’s surface detail information are generated in higher quantities by He irradiation than is the case with electron beams. As a result, low probe currents, on the order of one picoampere, can be used effectively to acquire HIM images. The use of small He ion beam currents is also necessary to minimize damage to the sample. However, such low current HIM images are often noisy, and important background structures may not be easily discernible due to the weak signal.

**Fig. 1:**
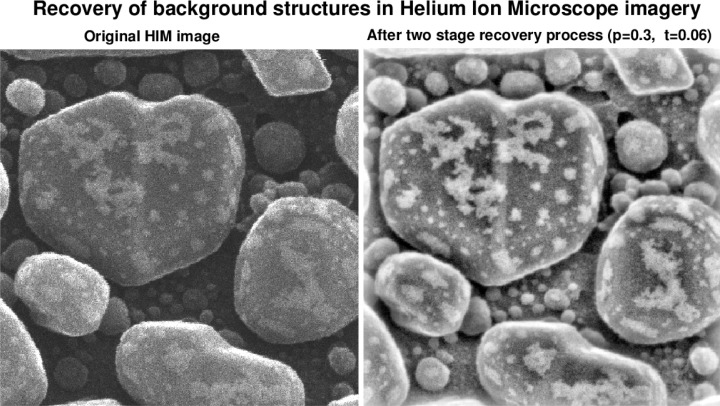
Two stage recovery process in original 600 nm field of view HIM image of Au-decorated gold on carbon sample, results in a companion image displaying significant background structural detail that is not readily apparent in the original image.

This paper discusses a two-step enhancement technique that can significantly improve this situation. As shown in Fig. 1, this technique results in a companion enhanced image in which deep background structural details are recovered, while noise is significantly reduced with minimal loss of image sharpness. Previous work on denoising HIM imagery [1], did not address the background recovery problem. While limited success was achieved in more recent work on forensic latent fingerprint enhancement [2], the present results, involving an entirely different class of images, could not be anticipated and had to be discovered independently.

**Fig. 2:**
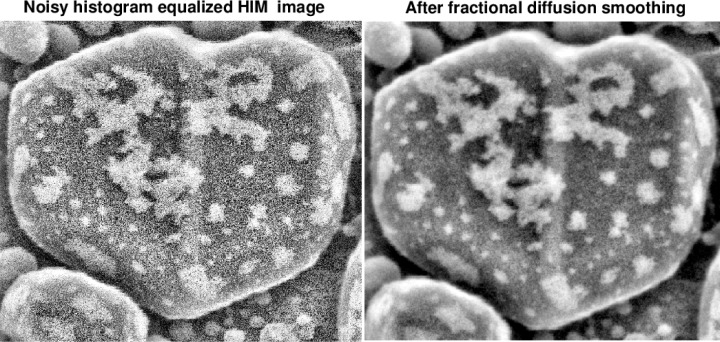
Two stage recovery process involves preliminary adaptive histogram equalization in original HIM image (left). This enhances background information but significantly amplifies noise. Second stage (right) applies versatile fractional diffusion smoothing that can filter out noise while preserving delicate morphological details.

**Fig. 3:**
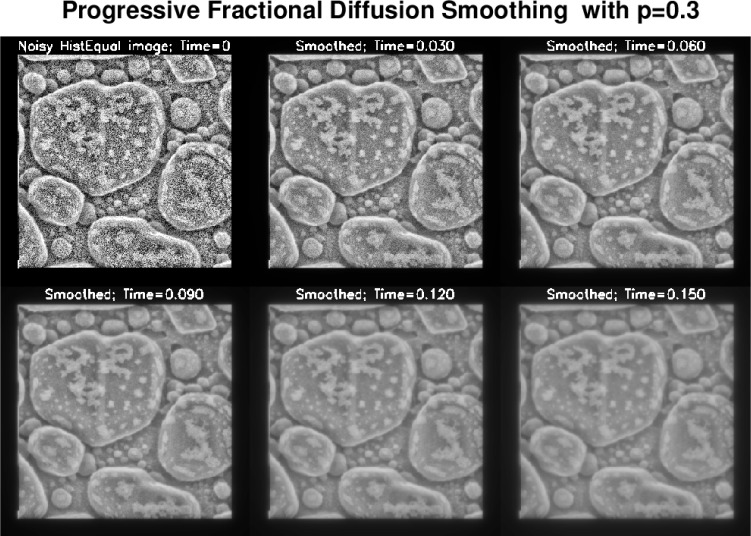
Second stage in recovery process is based on obtaing a sequence of progressively smoother images, by applying a low exponent fractional diffusion equation to the noisy histogram equalized HIM image. Considerable flexibility is provided by the possibility of choosing both the exponent *p* and the time *t* in Eq. (3,4). Here, with *p* = 0.3, the smoothed image at *t* = 0.06 is considered visually optimal. Other combinations of *p* and *t* may also yield useful results.

**Fig. 4:**
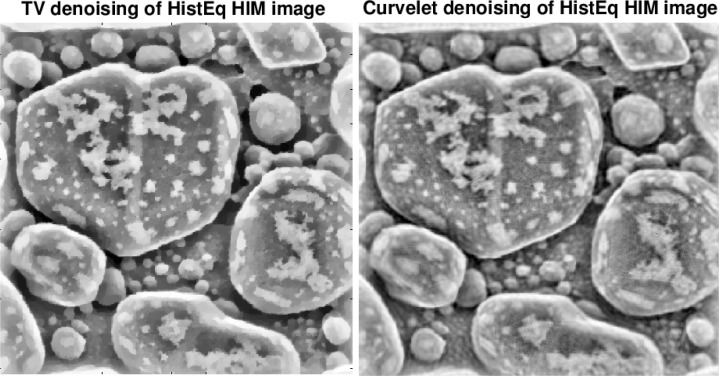
Split Bregman Total Variation denoising (left) generally does not preserve small-scale surface detail with sufficient fidelity. Denoising using Curvelet Thresholding (right) is time-consuming, and can generate misleading artifacts. Here, spurious criss-crossing lines in Curvelet image become clearly visible under magnification, when viewed on high resolution monitors.

The two stage approach used here involves a preliminary image-specific *adaptive histogram equalization* of the given HIM image [11]. Such equalization enhances background information while significantly magnifying noise, and is not generally advisable with noisy data. However, at the second stage, an effective and easy to use *progressive* denoising technique, based on low exponent Lévy fractional diffusion smoothing, can be successfully applied to this histogram equalized image with magnified noise. As may be inferred from Figs. 2 and 3, such Lévy smoothing can be fine-tuned interactively so as to preserve the detailed surface morphology of the sample. Despite many parameter trials, comparable fidelity to surface detail was not found feasible with some other better-known denoising techniques, such as methods based on minimizing the image ‘total variation’ norm [6], [8], or methods based on thresholding curvelet transforms [13], [14]. This is illustrated in Figure 4.

The ability to explore efficiently in parameter space and visually select the best result, is the key to successful digital image enhancement. There is no single set of parameters that will be useful in all cases, nor is there an automatic way of selecting the best set of parameters. Only a trained and experienced human analyst can locate useful parameter values, and determine the proper amount of fine tuning that best displays the information sought. The paper includes software routines, written in *Interactive Data Language* (IDL) [7], that can be used to perform the above two separate image processing tasks.

## 2. Preliminary Enhancement of HIM Image Using ‘IDLAdapt.pro’

Histogram equalization is a useful enhancement technique for images where significant information is suspected of being hidden in dark regions. A complete discussion of this technique is given in [11], together with examples illustrating the advantages of the more sophisticated *adaptive* formulation. However, in practice, usefully recovered background information is often obscured by the amplification of the accompanying noise, and the resulting improvements may not be particularly helpful without additional intervention.

**Fig. 5:**
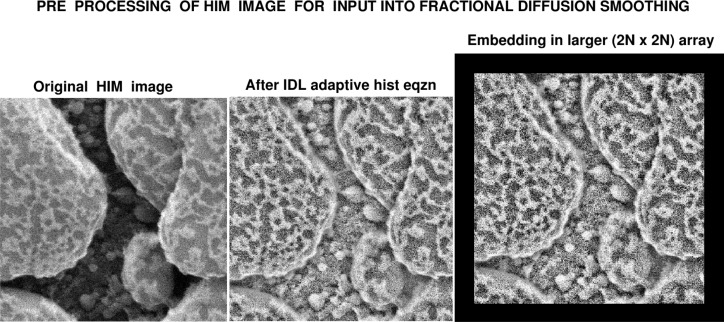
In Sec. 7, routine IDLAdapt.pro uses zero padding after histogram equalization, to produce larger embedded image, prior to input into progressive fractional diffusion routine IDLLevy.pro.

The routine *IDLAdapt.pro* listed in Sec. 7, is to be applied to an 8 bit greyscale TIFF image, which may be rectangular. The routine identifies and prints the dimensions of that image, performs adaptive histogram equalization, and displays the equalized image. Next, the equalized image is embedded in a zero padded larger square array of even dimension specified by the user. That dimension should typically be between fifteen and twenty percent greater than the larger side of the input image. The zero padded equalized 8 bit greyscale TIFF image is returned in the file ‘idlsharp.tiff’. This process is illustrated in Fig. 5.

## 3. Progressive Low Exponent Lévy Fractional Diffusion Smoothing

Given a noisy image *f* (*x*, *y*), the smoothing procedure results from solving an initial value problem for a special type of diffusion equation, with the image *f* (*x*, *y*) as initial data. Such smoothing is applied to the whole image, and not just to a selected portion of the image. With fixed *p* with 0 < *p* ≤ 1, consider the linear fractional diffusion initial value problem in *L*^2^(*R*^2^),

$$w_{t}=-{\left(-\Delta \right)}^{p}w,t>0,\;w(x,y,0)=f(x,y),$$ (1)

where Δ denotes the 2D Laplacian. This reduces to the classical heat conduction equation when *p* = 1. However, our smoothing procedure uses values of *p* ≪ 1, such as *p* = 0.2, for example. Define the 2D Fourier transform of the image *f* (*x*, *y*) by

$$ℱ\{f\}=\hat{f}(\xi ,\eta )\equiv \int_{R^{2}}f(x,y)\exp\{-2\pi i(\xi x+\eta y)\}dxdy.$$ (2)

Eq. (1) has the unique Fourier domain solution

$$\hat{w}(\xi ,\eta ,t)=\exp\{-t{\left[{\left(2\pi \xi \right)}^{2}+{\left(2\pi \eta \right)}^{2}\right]}^{p}\}\hat{f}(\xi ,\eta ),\;t>0,$$ (3)

from which *w* (*x*, *y*, *t*) can be found by inverse Fourier transformation

$$w(x,y,t)=\int_{R^{2}}\exp\{2\pi i(\xi x+\eta y)\}\exp\{-t{\left[{\left(2\pi \xi \right)}^{2}+{\left(2\pi \eta \right)}^{2}\right]}^{p}\}\hat{f}(\xi ,\eta )d\xi d\eta .$$ (4)

As is evident from Eq. (4) *w* (*x*, *y*, *t*) becomes increasingly smoother as *t* increases. However, for small *p*, and over a short time interval, the smoothed image may be expected to retain many of the essential features present in the initial data *f* (*x*, *y*).

In Eq. (3), the function

$$\hat{h}(\xi ,\eta ,t)=\exp\{-t{\left[{\left(2\pi \xi \right)}^{2}+{\left(2\pi \eta \right)}^{2}\right]}^{p}\},\;t>0,$$ (5)

is the Fourier transform of the Green’s function for the linear fractional diffusion equation in Eq. (1). For each fixed *t* > 0, the function in Eq. (5) is also the Fourier transform of an *isotropic Lévy stable probability density function* with exponent 2*p*, [12]. When *p* = 1, Eq. (5) corresponds to a Gaussian distribution. For *p* ≪ 1, Eq. (5) corresponds to a heavy-tailed density in physical (*x*, *y*) space. Unlike a Gaussian, that Lévy density is not known in closed form in the physical variables (*x*, *y*), and it has infinite mean and infinite variance.

Smoothing an image by convolution with a Gaussian is equivalent to using *p* = 1, and solving the heat conduction equation in Eq. (1). The significance of Lévy stable fractional diffusion smoothing with *p* ≪ 1 can be inferred from Eq. (3). Clearly, attenuation of high frequency information, corresponding to large (|*ξ*| + |*η*|), is dramatically more severe when *p* = 1, than it is when *p* = 0.1 for example.

## 4. FFT Lévy Fractional Diffusion Smoothing Using ‘IDLLevy.pro’

As illustrated in Fig. 5, the smoothing software routine *IDLLevy.pro*, listed in Sec. 7, assumes a zero padded square input image of even dimension, and returns a square smoothed image of the same size. The smoothed image can subsequently be cropped to the original size.

Given the 2*N* × 2*N* pixel image *f* (*x*, *y*) as initial data, ‘IDLLevy.pro’ computes the solution *w* (*x*, *y*, *t*) in Eq. (1) at any given *t* > 0, by using the forward and inverse FFT to implement the operations in Eq. (3) and Eq. (4) respectively. In order to render mathematical formulae more transparent, we use the same notation, $\hat{f}(\xi ,\eta )$, for both discrete and continuous Fourier transforms. In the discrete FFT case, the frequencies 2*πξ* and 2*πη* are understood to be integer-valued and to range from −*N* to *N*. After selecting a tentative maximum smoothing time *T_max_* at which to terminate the smoothing process, Eq. (4) can be evaluated at finitely many intermediate times 0 = *t*_0_ < *t*_1_ < *t*_2_ < *t*_3_ < *… = T_max_*, to create *a suite of progressively smoother images*. In ‘IDLLevy.pro’, a total of six images are displayed at times *t_m_* = {(*m*−1)*T_max_}/5, *m* = 1,…,6. The first image is the original unsmoothed IDL Histogram image, while the sixth image is the smoothest image at the final time *T_max_*. A user may select an image at some *t_m_* ≤ *T_max_* as the optimal image, or may elect to try a different value of *T_max_*. The routine ‘IDLLevy.pro’ is applied as follows:

- At the prompt, for the Lévy exponent *p*, a value between 0.1 and 0.4 is a good first choice, with the larger value reserved for very noisy images. As a rule, values ≤ 0.2 should be explored prior to choosing a larger *p*. For final time of smoothing *T_max_*, a number between 0.1 and 0.3 should be entered as a good first choice. Exploring several values of *T_max_* is likewise quite useful.
- For each choice of *T_max_*, the associated suite of six progressively smoother images is computed and displayed in a matter of a few seconds. The user is then prompted to select the optimal smoothed image by entering a picture number between 2 and 6. Several trial choices can be explored. For each trial selection of optimal image, the original unsmoothed image and the selected optimal image are displayed side by side.
- When a final selection is made, one exits ‘IDLLevy.pro’ by entering the number −1. The suite of six progressively smoother images corresponding to the last choice of *T_max_*, is in the file ‘LevyEvol.tiff’. The final selection of optimal image in that suite is now in the file ‘Levysmooth.tiff’.

## 5. Some Examples of HIM Image Enhancement

**Fig. 6:**
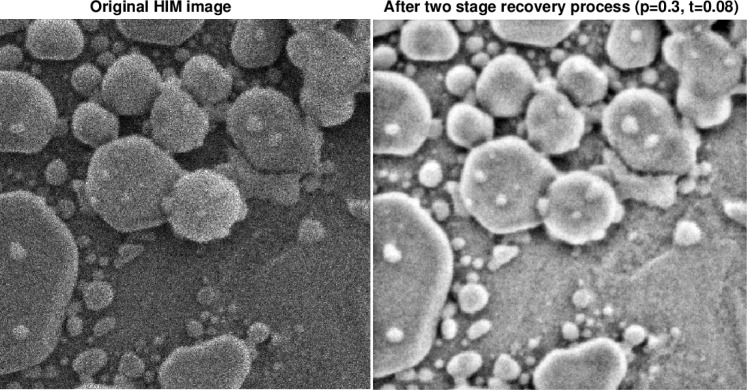
Two stage recovery in original 300 nm field of view HIM image of gold on carbon sample.

Figures 6 through 9 illustrate the kind of improvements that can be obtained using the above IDL software routines. Note the small values of *t* in these figures.

## 6. Drift-Corrected Composition of Multiple Fast Scan Frames

A NIST-developed image composition technique for scanning charged-particle microscopy is discussed in [3–5]. This methodology is based on super-fast acquisition of a large number of image frames. Due to the small beam currents generally used in scanning particle beam microscopy, these individual frames are inherently very noisy, but exhibit significantly less drift-related distortions. The drift correction takes place after finding the center of each frame, properly aligning these frames, and adding them together into a single image. Such composed images contain much less noise and exhibit significantly less blur and deformation than do images obtained by traditional slow scan methods, or images obtained by simply adding together fast image frames without compensating for drift. To improve repeatability of HIM and SEM images, this technique must be used with the minimum number of fast images, which limits the achievable SNR. In many cases, the resulting composed image is still somewhat noisy and some type of noise processing may be beneficial.

**Fig. 10:**
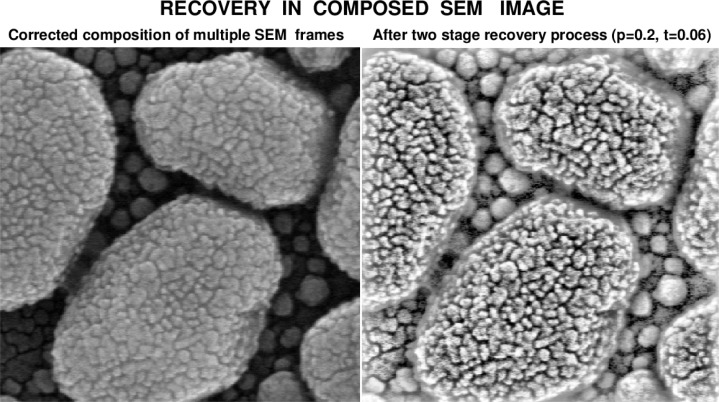
Two stage recovery in composed SEM image of platinum decorated gold on carbon sample, with a field of view of 441 nm.

**Fig. 11:**
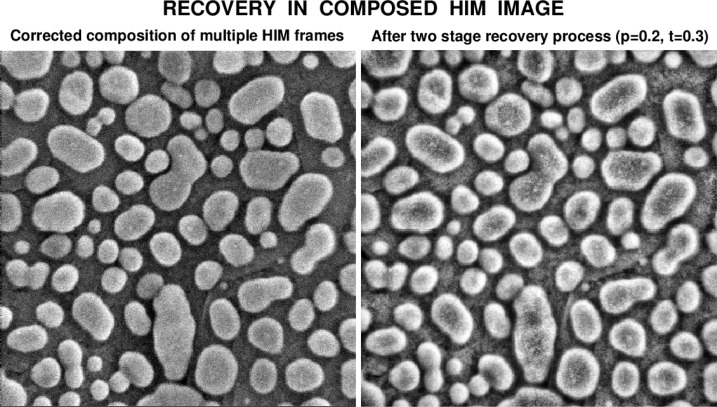
Two stage recovery in composed HIM image of gold on carbon sample, with a field of view of 500 nm.

As shown in Fig. 10, useful improvements of such composed SEM imagery are possible using the techniques in the present paper. Additionally, improvements to composed HIM imagery are also possible as shown in Fig. 11. The left image in Fig. 11 is a composition of 10 fast image frames. The NIST processed right image in Fig. 11 shows new details, such as augmented faint greyscale transitions at the tops of the gold grains, as well as very fine structures near these tops. Such details can hardly be seen initially in the original left image; however, they become discernible in the original image after studying the companion NIST-enhanced image on the right.

**Fig. 12:**
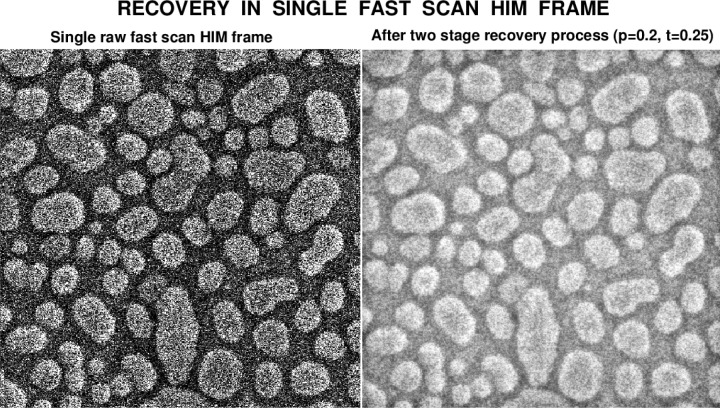
Two stage recovery in single raw fast scan HIM image of gold on carbon sample, with a field of view of 500 nm.

Improvements to single raw HIM image frames are also possible as shown in Fig. 12. One can recognize features in the enhanced right image in Fig. 12 that might easily have been dismissed as noise in the raw image, rather than actual information already present in that raw image.

Because the very finely focused helium ion beam in HIM imaging readily mills the gold particles in Fig. 12, long image acquisition times are not possible without substantially altering the sample. The best solution is the acquisition of a set of fast images that can be composed into a single image, yet one that does not show significant sample modification. This often leads to inherently noisy images. However, this can be remedied using the two-step process discussed in this paper, which results in less noisy images with more perceptible fine details.

## 7. Two IDL Routines for HIM Image Enhancement

The two software routines listed below were used to produce the enhanced images shown in the figures.

**IDL ROUTINE FOR ADAPTIVE HISTOGRAM EQUALIZATION**


;pro-file IDLAdapt.pro
;APPLY BY TYPING ‘.run IDLAdapt’ in IDL
;ASKS USER TO PROVIDE INPUT 8 bit GREYSCALE TIFF IMAGE
;IDENTIFIES AND PRINTS X size and Y size of INPUT TIFF IMAGE
;PERFORMS IDL ADAPTIVE HISTOGRAM EQUZN ON INPUT TIFF IMAGE
;DISPLAYS EQUALIZED IMAGE AND ASKS USER FOR DESIRED SIZE OF
;LARGER ZERO PADDED SQUARE EMBEDDED EQUALIZED IMAGE
;PADDED SIZE SHOULD BE EVEN INTEGER ABOUT 20 LARGER THAN INPUT SIZE
;RETURNS ZERO PADDED EQUALIZED IMAGE IN FILE "idlsharp.tiff"
file1=’ ’
read,’enter filename: ’,file1
image=read_tiff(file1)
ssz=size(image)
x1=ssz(1)
y1=ssz(2)
print,’the size of this array is ’,x1,’ ’,y1
window,0,xsize=x1,ysize=y1
image=adapt_hist_equal(image)
tvscl, image
x2=0
y2=0
read,"enter size of DESIRED ZERO PADDED SQUARE image: ",x2
  if (x2 le max([x1,y1])) then begin
  print,’DESIRED IMAGE TOO SMALL’
  goto,finishup
  endif
y2=x2
hnx2=(x2-x1)/2
hny2=(y2-y1)/2
image2=bytarr(x2,y2)
image2(hnx2:hnx2+x1-1,hny2:hny2+y1-1)=image
window,1,xsize=x2,ysize=y2
tvscl, image2
write_tiff, ’idlsharp.tiff’, image2
finishup:
end

**IDL ROUTINE FOR LÉVY FRACTIONAL DIFFUSION SMOOTHING**


;pro-file IDLLevy.pro
;APPLY BY TYPING ‘.run IDLLevy’ in IDL
;ASKS FOR INPUT ZERO PADDED 2Nx2N 8bit GREYSCALE TIFF IMAGE
;IDENTIFIES AND PRINTS SIZE OF INPUT IMAGE
;RETURNS USER SELECTED OPTIMAL SMOOTH IMAGE IN FILE ‘Levysmooth.tiff’
;RETURNS PROGRESSIVELY SMOOTHER 6 IMAGE SUITE IN FILE ’LevyEvol.tiff’
file1 = ’ ’
sz= ’ ’
plev= ’ ’
time= ’ ’
read,’enter filename (e.g. idlsharp.tiff): ’, file1
image=read_tiff(file1)
ssz=size(image)
x1=ssz(1)
y1=ssz(2)
print,’the size of this array is ’,x1,’ ’,y1
sz=x1
read,’enter Levy exponent value p, (e.g. 0.2):’, plev
read,’enter final time of smoothing, (e.g. 0.3 ): ’, time
close,1
openu,1,file1
a = assoc(1,bytarr(sz,sz,/nozero))
B=a(0)
SB=Size(B,/dimensions) & N=SB[0] & M=SB[1]
u=(Findgen(N)-N/2)#Replicate(1,M)
v=(Findgen(M)-M/2)##Replicate(1,N)
window,0,xsize=1800, ysize=1200
DEVICE, DECOMPOSED=0
LOADCT,0
B=Reverse(B,2)
BB=CONGRID(B,600,600)
p= plev
r2=u*u+v*v
r2p=(r2 ^ p)
dt=time/5.0
AF=FFT(B)
AFS=Shift(AF,N/2,M/2)
TV, BB, 0
XYOUTS,300,1165,’IDL AdaptHistEq; Time=0’,Alignment=0.5,$
CHARSIZE=3.25, CHARTHICK=4.0, /DEVICE, color=255
  for i=1,5 do begin
  h=exp(-i*dt*r2p)
  AFH=h*AFS
  BH=Abs(FFT(AFH,/Inverse))
  BBH=CONGRID(BH,600,600)
  TV, BBH, i
    if (i le 2) then begin
    XYOUTS, 300+i*600, 1165, $
    ’Smoothed; Time=’+strsub(si(i*dt),0,4),$
    Alignment=0.5, CHARSIZE=3.25, CHARTHICK=4.0, /DEVICE, color=255
    endif
    if (i ge 3) then begin
    XYOUTS, 300+(i-3)*600, 570, $
    ’Smoothed; Time=’+strsub(si(i*dt),0,4),$
    Alignment=0.5, CHARSIZE=3.25, CHARTHICK=4.0, /DEVICE, color=255
    endif
  sidt= si(i*dt)
  endfor
GB=TVRD(0,0,1800,1200)
write_tiff, ’LevyEvol.tiff’, GB
i=0
read,’enter picture number [2..6 to select image, or -1 to quit]: ’,i
  while (i ne -1) do begin
  print,’picture number: ’,i
  j=i-1
  h=exp(-j*dt*r2p)
  AFH=h*AFS
  BH=Abs(FFT(AFH,/Inverse))
  BB=CONGRID(B,1200,1200)
  BBH=CONGRID(BH, 1200,1200)
  window,0,xsize=2400, ysize=1200
  TV, BB, 0
  XYOUTS, 600,1130, ’Unsmoothed; Time=0’, Alignment=0.5,$
  CHARSIZE=9.5, CHARTHICK=5.0, /DEVICE, color=255
  TV, BBH, 1
  XYOUTS, 1800,1130, ’Smoothed; Time=’+strsub(si(j*dt),0,4),$
  Alignment=0.5, CHARSIZE=9.5, CHARTHICK=5.0, /DEVICE, color=255
  read,’enter picture number (-1 to quit): ’,i
  endwhile
write_tiff, ’Levysmooth.tiff’, BH
close,1
end

## Figures and Tables

**Fig. 7:**
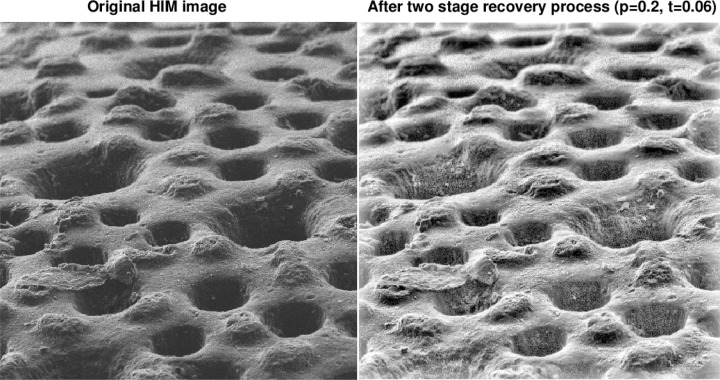
Two stage recovery in original 45 μm field of view HIM image of radiolaria sample.

**Fig. 8:**
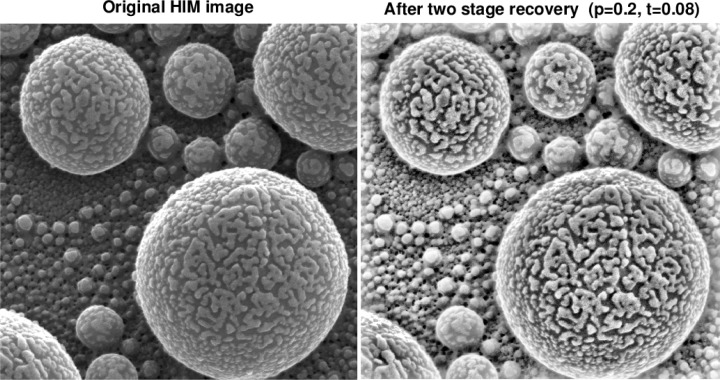
Two stage recovery in original 1.5 μm field of view HIM image of Au decorated tin ball sample

**Fig. 9:**
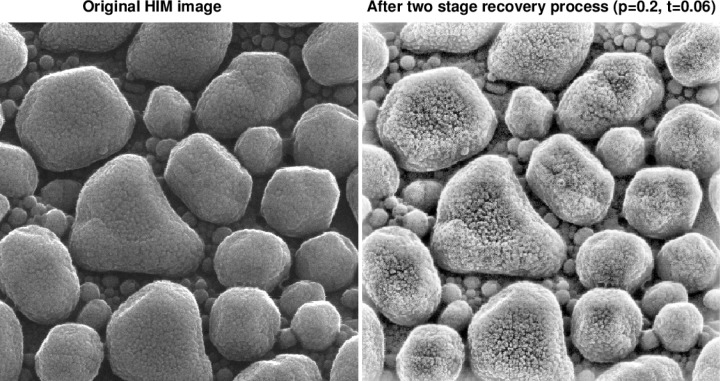
Two stage recovery in original 1 μm field of view HIM image of Auc7 sample.
